# Supplementary material for: The Role of Distinctive Sphingolipids in the Inflammatory and Apoptotic Effects of Electronegative LDL on Monocytes
Source: Biomolecules. 2019 Jul 24;9(8):300. doi: 10.3390/biom9080300 (PMC6722802; doi:10.3390/biom9080300)
Supplement: Supplementary file 1 [file biomolecules-09-00300-s001.pdf]

Supplementary Figure 1

Fig 1 A

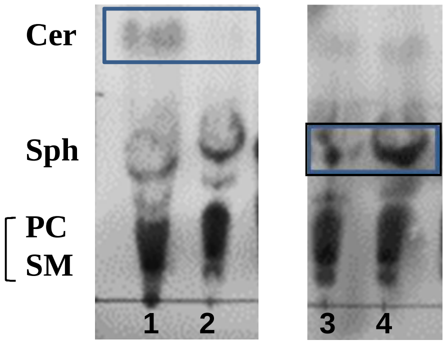

Fig 1 B

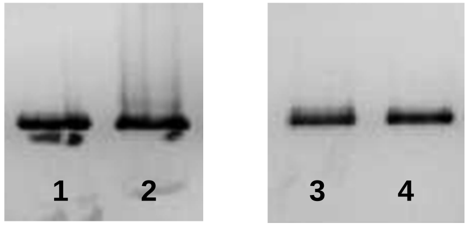

Supplementary Fig 1 . Modification of LDL by Sph enrichment and SMase treatment (n=4)
1 A). Representative thin-layer chromatography. 1-SMase-LDL(10); 2-Smase-LDL (0); 3- Sph-LDL (0); 4- Sph-LDL (10)
1 B) Representative gradient gel electrophoresis. 1-SMase-LDL(0); 2-Smase-LDL (10); 3- Sph-LDL (0); 4- Sph-LDL (10)

Supplementary Table 1. Monocyte apoptosis in the absence (blank) or presence of inhibitors (all at 10 µM)

% cells	Blank	MAPP	DMS	CPZ
Early apoptosis	1.64 ± 1.15	1.06 ± 1.18	5.93 ± 3.87	1.20 ± 0.50
Late apoptosis	2.95 ± 0.95	4.75 ± 1.42	10.78 ± 3.10*	4.43 ± 2.82
Total apoptosis	4.59 ± 2.46	5.82 ± 3.29	16.71 ± 4.92*	5.63 ± 3.29

*vs blank, $p<0.05$, (n=5).
